# Exercise-induced pseudo-ischaemic electrocardiographic changes in a female with concave-shaped chest wall

**DOI:** 10.1093/ehjcr/ytae123

**Published:** 2024-03-12

**Authors:** Andrea Sonaglioni, Gian Luigi Nicolosi, Michele Lombardo

**Affiliations:** Division of Cardiology, Istituto di Ricovero e Cura a Carattere Scientifico (IRCCS) MultiMedica, Via San Vittore 12, 2023 Milan, Italy; Division of Cardiology, Policlinico San Giorgio, Via Agostino Gemelli 10, 33170 Pordenone, Italy; Division of Cardiology, Istituto di Ricovero e Cura a Carattere Scientifico (IRCCS) MultiMedica, Via San Vittore 12, 2023 Milan, Italy

A 54-year-old woman (body surface area 1.55 m^2^, body mass index 20.2 kg/m^2^), with mild dyslipidaemia and family history of coronary artery disease (CAD), was referred to our outpatient cardiology clinic to perform an exercise stress testing (EST). She did not have any previous cardiovascular event nor cardiac symptoms. On physical examination, she had a mild degree of anterior chest wall deformity, as non-invasively assessed by the modified Haller index (MHI)^1^ value (equal to 2.6) (*Figure 1A* and *B*). Resting electrocardiogram showed sinus rhythm with non-specific ST-T repolarization abnormalities in the anterolateral leads (*Figure 1C1*). Exercise stress testing revealed marked downsloping ST-segment depression in the anterolateral leads, maximum 2.5 mm in V5 at peak exercise (*Figure 1C2*), with slow normalization during the recovery period. Peak exercise blood pressure was 160/80 mmHg and the patient did not manifest any symptom. Computed tomography coronary angiography confirmed a mild increase in the Haller index (equal to 2.6) (*Figure 1D*) and documented integrity of all coronary arteries (*Figure 1E–L*). Transthoracic echocardiography (TTE) showed normal cardiac chambers cavity size, normal biventricular systolic function, and a bileaflet mitral valve prolapse (MVP) with mild mitral regurgitation (*Figure 1M*). Global longitudinal strain was moderately reduced (−16.6%), due to impairment of myocardial deformation in basal and mid segments (*Figure 1N*).

**Figure 1:**
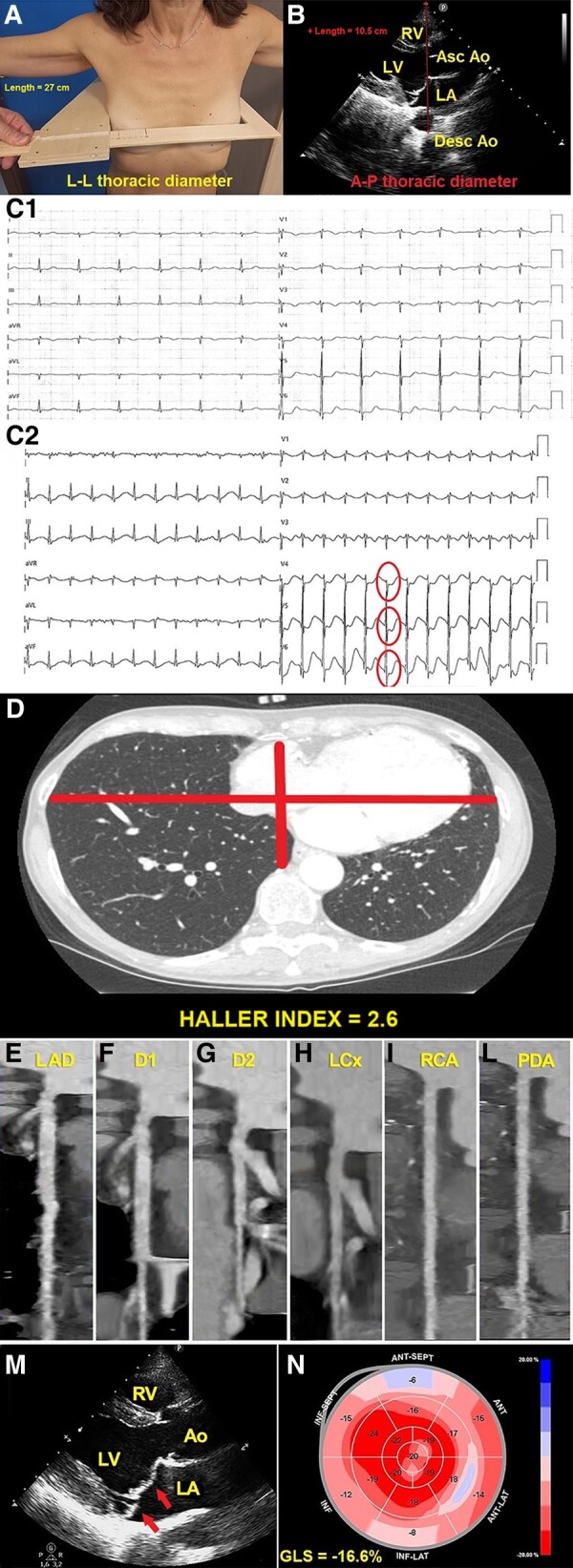
(*A*) L-L thoracic diameter, measured at end inspiration with the individual in the standing position and with open arms, by using a rigid ruler in centimetres coupled to a level (the measuring device), placed at the distal third of the sternum, in the point of maximum depression of the sternum. L-L, latero-lateral. (*B*) A-P thoracic diameter, obtained with the individual in the left-lateral decubitus position, during conventional transthoracic echocardiography, by placing a 2.5 mHz transducer near the sternum in the left third or fourth intercostal space, to obtain a parasternal long-axis view, and measuring at end inspiration the distance between the true apex of the sector and the anterior surface of the vertebral body. The vertebral body is identified by using, as a reference point, the posterior wall of the descending thoracic aorta, visualized behind the left atrium. Ao, aorta; A-P, antero-posterior; Asc, ascending; Desc, descending; LA; left atrium; LV, left ventricle; MHI, modified Haller index; RV, right ventricle. (*C1*) Resting electrocardiogram showing sinus rhythm with mild right ventricular conduction delay and non-specific ST-T repolarization abnormalities in the anterolateral leads. (*C2*) Peak-exercise electrocardiogram showing marked downsloping ST-segment depression in the anterolateral leads V4–V6 (circles) (maximum 2.5 mm in V5). (*D*) Axial computed tomography scan assessing conventional Haller index, the ratio of chest transverse diameter over the distance between the sternum and spine. (*E–L*) Computed tomography coronary angiography: curved planar reformation images of left anterior descending artery (*E*), D1 (*F*), D2 (*G*), left circumflex artery (*H*), right coronary artery (*I*), and posterior descending artery (*L*), with no evidence of obstructive coronary artery disease. D1, first diagonal branch; D2, second diagonal branch; LAD, left anterior descending artery; LCx, left circumflex artery; PDA, posterior descending artery; RCA, right coronary artery. (*M*) Parasternal long-axis view demonstrating bileaflet mitral valve thickening and prolapse (arrows). Ao, aorta; LA, left atrium; LA, left ventricle; RV, right ventricle. (*N*) Global longitudinal strain bull's eye plot assessed by strain echocardiographic imaging. This example shows a moderate impairment in global longitudinal strain magnitude (−16.6%), secondary to a significant reduction in left ventricular basal strain values, represented as light red, light pink, and pale and/or light blue. GLS, global longitudinal strain; LV, left ventricular.

In the present case, the continuous mechanical stress perpetuated by the anterior chest wall deformity might have contributed to the peak-exercise ‘pseudo-ischaemic’ electrocardiogram (ECG) abnormalities, likely induced by cardiac compression, rotation, and tilting with dyssynchrony of myocardial segments. This mechanical hypothesis is further supported by the evidence that, in clinical practice, it is not uncommon to encounter patients with various degrees of anterior chest wall deformity, which are diagnosed with MVP on TTE, ‘pseudo-ischaemic’ ECG changes on EST, and finally impaired myocardial strain at the mid-basal level on strain echocardiographic imaging.

The association between anterior chest deformity and MVP might also have a developmental^2^ or genetic basis.^3^

A preliminary chest shape assessment should be implemented in clinical practice for better identifying the individuals with increased probability of false-positive EST results.

## Data Availability

The data underlying this article will be shared on reasonable request to the corresponding author.
